# Supplementary material for: CRISPR/Cas9 targeted CAPTURE of mammalian genomic regions for characterization by NGS
Source: Sci Rep. 2019 Mar 5;9:3587. doi: 10.1038/s41598-019-39667-4 (PMC6401131; doi:10.1038/s41598-019-39667-4)
Supplement: Supplementary file 1 — CRISPR/CAS9 TARGETED CAPTURE OF MAMMALIAN GENOMIC REGIONS FOR CHARACTERIZATION BY NGS [file 41598_2019_39667_MOESM1_ESM.docx]

**CRISPR/Cas9 targeted CAPTURE of mammalian genomic regions for characterization by NGS**

Alexei Slesarev, Lakshmi Viswanathan, Yitao Tang, Trissa Borgschulte, Katherine Achtien, David Razafsky, David Onions, Audrey Chang, Colette Cote

**Supplemental Information**

Supplementary Note 1: Reconstruction of hamster chromosome 9 and 10……………………………….2

Supplementary Note 2: Reconstruction of hamster chromosome 7…………………………………………..5

Supplementary Note 3: Identification of Donor DNA Integration Breakpoints….……………………….7

Supplementary Note 4. Host|Vector and Vector|Vector fusions and evidence…………………………9

Supplementary Figures 9-12 …………………………………………………………………………………………………….13

**Supplementary Note 1**: Reconstruction of hamster chromosome 9 and 10

Using the GCA_000448345.1 (Cgr1.0) assembly, we have filtered out scaffolds not belonging to chromosomes 9+10 from all unplaced scaffolds. It was done by mapping unplaced scaffolds against mouse chromosome 15 using BLAST with an E-value cutoff of 1X10^-5^ and an alignment length cutoff of ≥ 500 bp (see Supplementary Table 5). This resulted in 14 scaffolds (KE687004.1, KE687988.1, KE691351.1, KE688988.1, KE689850.1, KE691141.1, KE691439.1, KE686446.1, KE687468.1, KE690596.1, KE686185.1, KE686184.1, KE686186.1, KE686527.1) that covered the entire region of homology between hamster chromosome 9+10 and mouse chromosome 15^1^. Eight out of fourteen identified scaffolds (KE687004.1 KE687988.1 KE691351.1 KE688988.1 KE689850.1 KE691141.1 KE691439.1 KE686446.1) fit into a single 17.56 Mb scaffold_34 from the most recent CHO-K1 genome assembly CHOK1GS_HDv1^2^ (GenBank assembly accession GCA_900186095.1), while the remaining six scaffolds match multiple contigs in the CHOK1GS_HDv1 assembly. Based on the previously established homology of the chromosome 9+10 scaffolds to an internal region of the mouse chromosome 15^1^, we used Mauve aligner^3^ to order these scaffolds assuming their synteny with the mouse chromosome 15 region (mouse assembly GRCm38/mm10) and using the following parameters: match seed weight of 17, three spaced seed patterns, minimum LCB weight of 200, collinear mode. The output from Mauve in a backbone format was fed into the R package genoPlotR^4^ to visualize the synteny map between the hamster chromosome 9 and 10 scaffolds and mouse chromosome 15 (Supplementary Figure 1). The resulting sequence (consisting of ordered scaffolds totaling ~32.2 Mb) was used to analyze its segmented copy number for different cell lines with cnvkit^5^. Assuming a diploid sequence nature, a partial single-copy loss was observed after ~17.56 Mb (log2(1/2) = -1.0) in the case of the AE54SL cell line, while a four-copy gain was observed starting at the same location in the AD49ZG and AD49ZH cell lines (Supplementary Figure 1). Considering that Cgr1 scaffolds KE687004.1 KE687988.1 KE691351.1 KE688988.1 KE689850.1 KE691141.1 KE691439.1 KE686446.1 are assembled into one 17.56 Mb scaffold_34 in the CHOK1GS_HDv1 assembly, and that the copy number of this scaffold is unchanged across all cell lines tested, we have effectively split the combined ~32.5 Mb structure into two chromosome sequences: a ~17.5 Mb chromosome 9 and a ~14.7 Mb chromosome 10. The sequences reconstructed in this manner should be viewed as chromosome 9 and 10 backbones stripped of most repeats and likely some unique sequences not represented in the mouse genome.


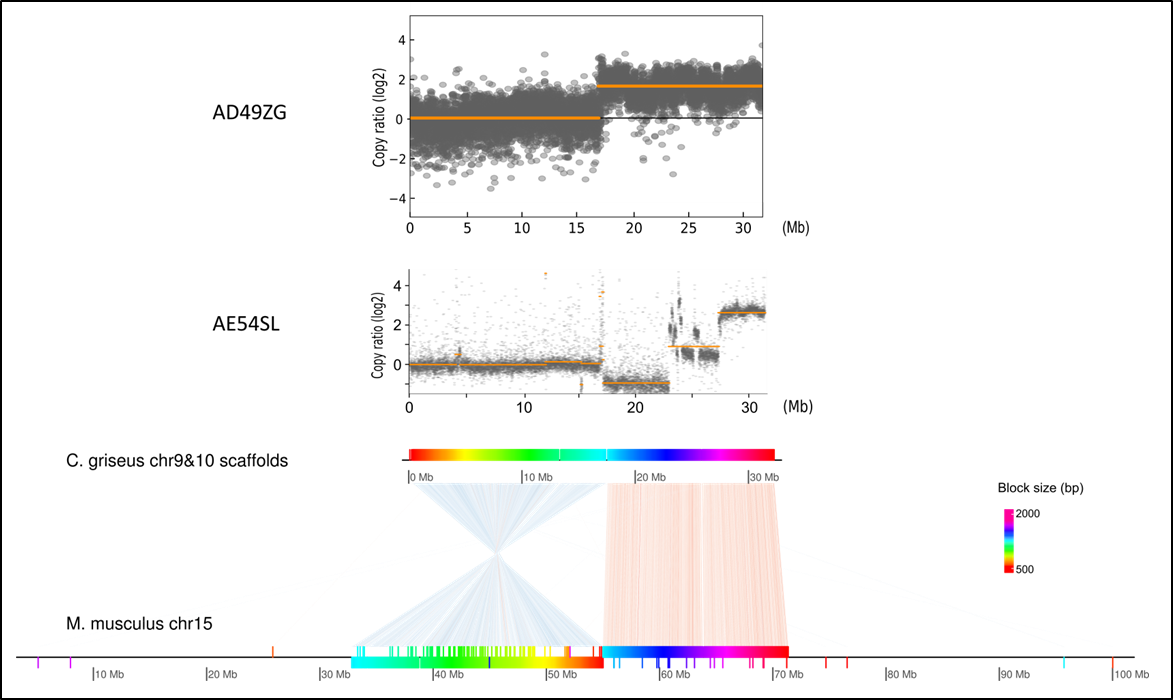


**Supplementary Figure 1**| Lower panel: mauve alignment of hamster chromosome 9+10 scaffolds to mouse chromosome 15. The strand indicates the orientation of the block with respect to the mouse chromosome and the segments are colored according to their lengths. The resulting mauve blocks were parsed and plotted by genoPlotR^4^. Middle panel: copy number variation along the combined sequence for the AE54SL cell line (datasets from four Illumina® Miseq WGS runs; data were plotted using CNVkit^5^). Top panel: copy number variation along the combined sequence for the AD49ZG cell line (HiSeq2500 WGS run).


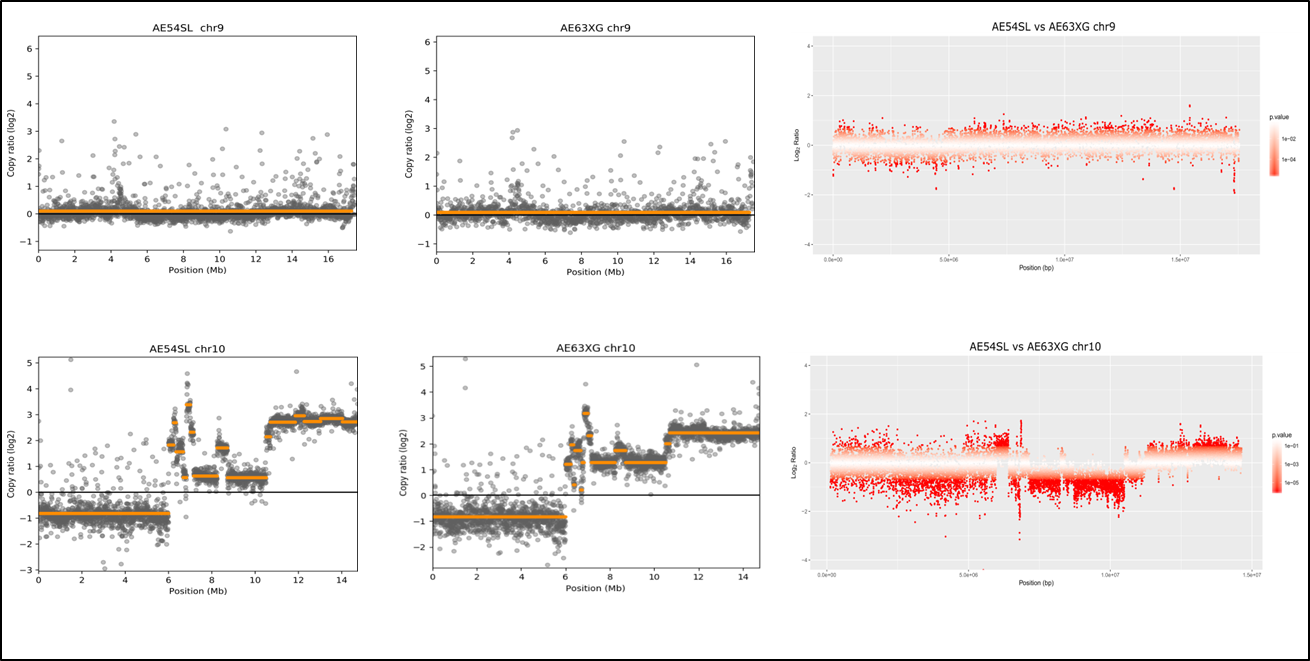


**Supplementary Figure 2**| Copy number variation of hamster chromosomes 9 and 10 in two cell lines detected using WGS data. AE63XG cells were used for transfection with pGMX plasmid to generate the AE54SL cell line. The first two columns show corresponding log2 chromosome coverages for individual genomes. The third column shows relative CNVs in each chromosomal region between the two genomes. The red color gradient represents log10 *p* calculated for each ratio^6^. Chromosome 9 shows no apparent structural variations and/or copy number changes in AE63XG or AE54SL; chromosome 10 is rearranged in the AE63XG genome and underwent even further structural changes and CNVs in the AE54SL cell line.

**Supplementary Note 2**: Reconstruction of hamster chromosome 7

Almost all of 1,007 hamster chromosome 7 scaffolds from the Cgr1.0 assembly (total contig length of 137 Mb) were mapped to the scaffold_4 ( 87.8 Mb), scaffold_12 (54.9 Mb), and scaffold_62 (2.41 Mb) sequences of the CHOK1GS_HDv1 assembly^2^ using BLAST with an E-value cutoff of 1X10^-5^ and an alignment length cutoff of ≥ 500 bp (see Supplementary Table 5). This finding suggests the latter three scaffolds constitute the entire backbone of the hamster chromosome 7. Mapping these three scaffolds with mauve aligner^3^ in collinear mode to mouse chromosomes 11, 12, 16, and 17 revealed substantial scaffold synteny with homologous parts of mouse chromosomes (Supplementary Figure 3) and suggested their following order in the hamster chromosome 7: scaffold_4, scaffold_62, scaffold_12.


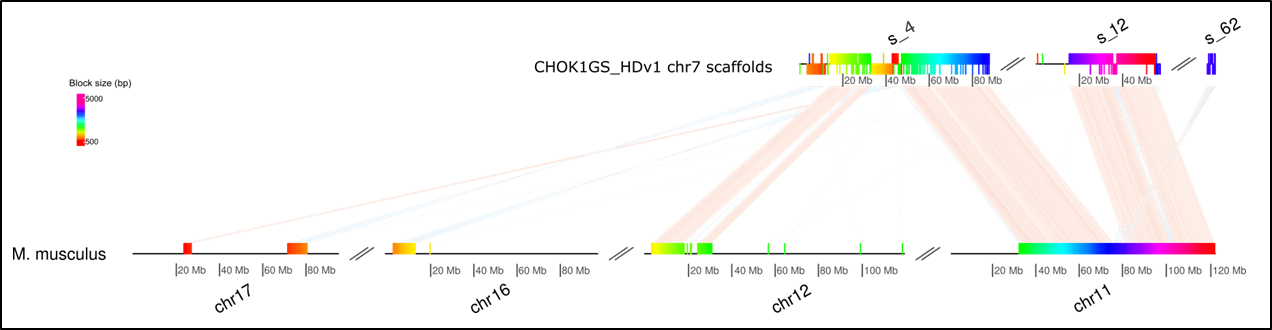


**Supplementary Figure 3**| Reconstruction of hamster chromosome 7. The backbone file was produced by mauve aligner with the following parameters: match seed weight of 17, three spaced seed patterns, minimum LCB weight of 200, collinear mode. The resulting mauve blocks were parsed and plotted by genoPlotR^4^.


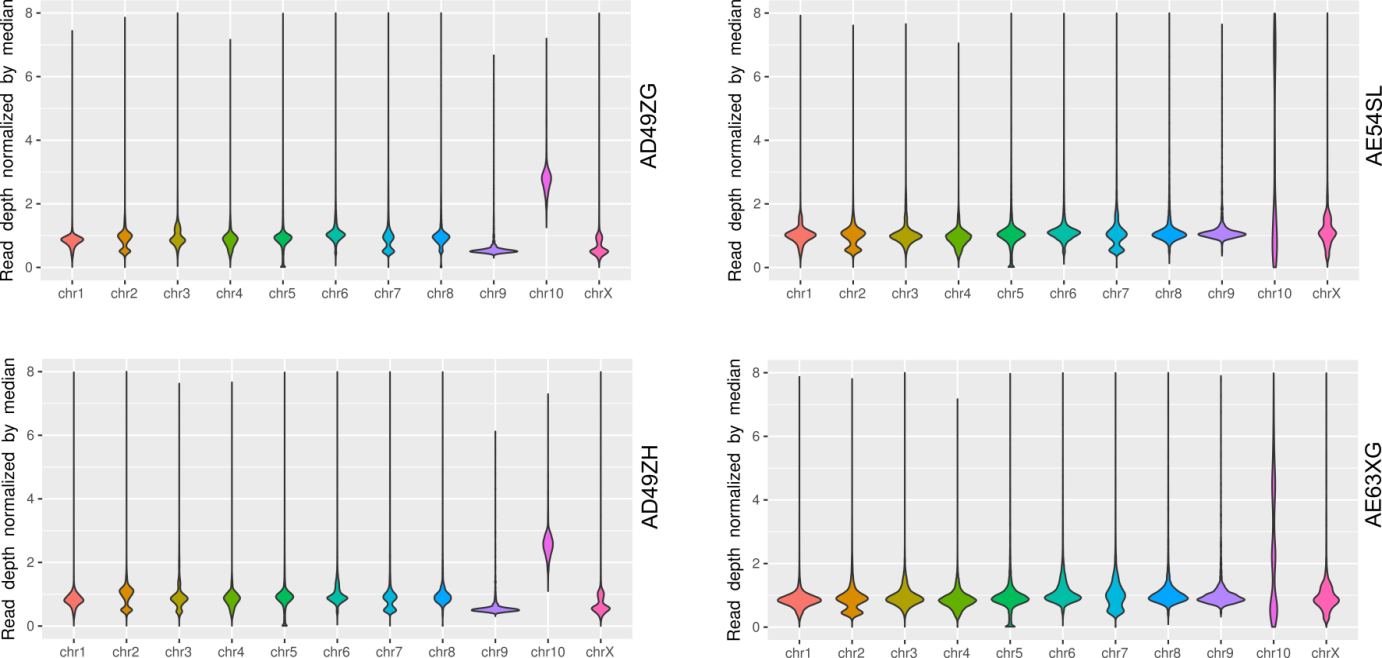


**Supplementary Figure 4**| Distribution of normalized read depth (dividing by the whole-genome median for each dataset), broken out by chromosome for each analyzed cell line. Chromosomes 2, 3, 7, 10, and X have CNV regions.

**Supplementary Note 3**: Identification of Donor DNA Integration Breakpoints

This workflow is based on Illumina paired-end reads and is meant to convey an approach that proved to be effective for discovering structural variation breakpoints in CHO cell lines involving donor (primarily vector) DNA. This approach seeks to discover breakpoints arising from both "unique" and duplicated/concatenated vector sequences. Therefore, it includes steps that attempts to capture mappings for read pairs that align to such duplicated sequences. The pipeline then uses those mappings to search for vector insertions and rearrangements among segmental duplications.

**An example workflow**.

The "$" denotes the command line prompt.

*Read mapping, sorting and indexing bam file*. We created a custom reference genome comprised of CHOK1 chromosomes, vector and *E. coli* fasta sequences as pseudochromosomes. Vector and *E. coli* genome sequences were appended to hamster chromosomes to compose a multi fasta reference genome. This custom CHOK1–Vector-E. coli reference genome is then used for aligning reads with BWA aligner. This step is meant to quickly identify donor DNA aligned discordant read pairs and split reads from a BAM file by parsing the BAM file for reads where one mate is aligned to a hamster chromosome and another to a donor sequence.

$ bwa mem -t <THREADS> <CUSTOM GENOME>.fasta ForwardReads.fq \ ReverseReads.fq | samtools view -uS - | samtools sort -@ <THREADS> \

-o <CUSTOM GENOME>.bam && samtools index <CUSTOM GENOME>.bam \

<CUSTOM GENOME>.bam.bai

# extracting discordant paired-end vector alignments

$ samtools view -F 4 -f 8 clone27.bam <DONOR DNA>|awk -F "\t" '{print $1}'| \ sort -u > DonorDiscordant.list

# creating a file with IDs of mapped split read pairs

$ samtools view <CUSTOM GENOME>.bam <DONOR DNA>|awk -F "\t" \

'{if (($6 ~ /S/) && ($7 ~ /chr/)) {print $1} }' | sort -u > DonorSplit.list

# extracting donor split read pairs and donor discordant pairs from the #dataset using bbmap

$ cat donorSplitReads.list donorDiscordantReads.list | sort -u > \ SplitDiscordantNames.list

$ filterbyname.sh in=ForwardReads.fq in2=ReverseReads.fq \ out=SplitDiscordantForward.fq out2=SplitDiscordantReverse.fq \ names=SplitDiscordantNames.list include=t

*Assembly of discordant and split-read sequences.* The assembly is performed in an effort to identify the exact nucleotide at which the integration breakpoint(s) occurred. For this, we assemble the corroborating discordant and split read sequences with SPades.

$ spades.py -t <THREADS> -k 33,55,77,127 –-pe1-1 SplitDiscordantForward.fq \ –-pe1-2 SplitDiscordantReverse.fq -o .

The Spades contigs are then aligned to the custom genome database with megablast:

$ blastn -query contigs.fa -db <CUSTOM GENOME>.fasta –task megablast -outfmt 6 \

-num_threads <THREADS> -max_target_seqs <N>

By adjusting blastn parameters, the alignment can be made more sensitive to ensure that all homology between the assembled breaktigs and the reference locus would be detected. As a consequence of the sensitive alignment setting used, there are frequently cases where the same sections of a breaktig align to multiple locations within the reference genome locus. This occurs, for example, when a breaktig contains a common repeat. In such cases the largest alignment is retained for a given section of the breaktig. All identified breakpoints were eventually visualized and verified in Ribbon^7^ (Supplementary Note 4). In general, using a GUI program for inspecting vector integration sites is not mandatory but is highly recommended because vector sequences often undergo complex rearrangements, that may still be missed by command line tools.

**Supplementary Note 4**: Chromosome|Donor and Donor|Donor fusions and evidence

All figures in this section are generated using Ribbon^7^ to visualize the vector integration events. The top multi-read view (A) in each figure is filtered to show only regions with at least 10 alignments; otherwise, all reads that have alignments at the relevant breakpoints are shown including all of their other alignments everywhere else in the genome. In each figure, one of the supporting reads is selected for (B) that clearly showcases all of the variants involved in the vector chromosome integration.


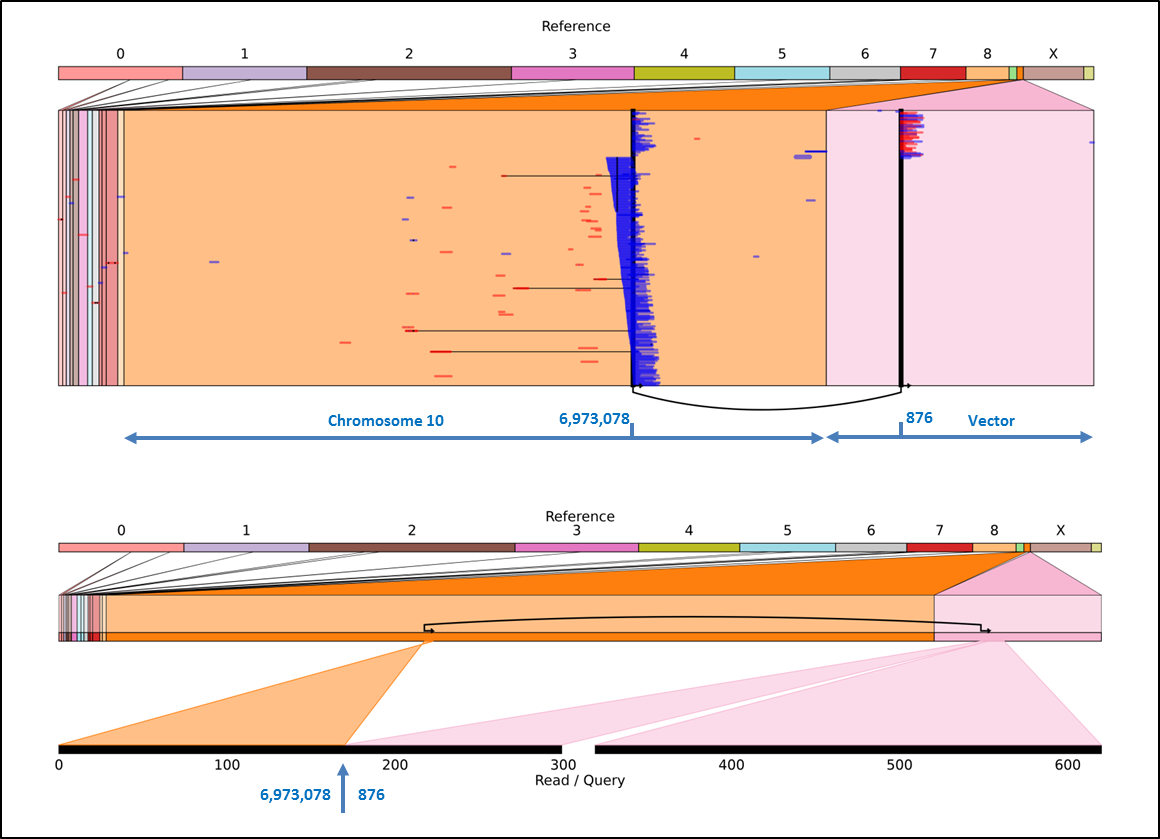


**Supplementary Figure 5**| Ribbon plot of Illumina bwa mem alignment from the chromosome 10|pGMX junction in the AE54SL cell line (RGEN-D protocol). The top panel is the multi-read view of the region involved in the vector integration created by a single variant. The structural variant is shown as a connecting line underneath in black and paired-end alignments as blue (forward) and red (reverse) where direction is with respect to the alignments at the breakpoint. Bottom panel shows an example of the read pair supporting the chromosome|vector junction.


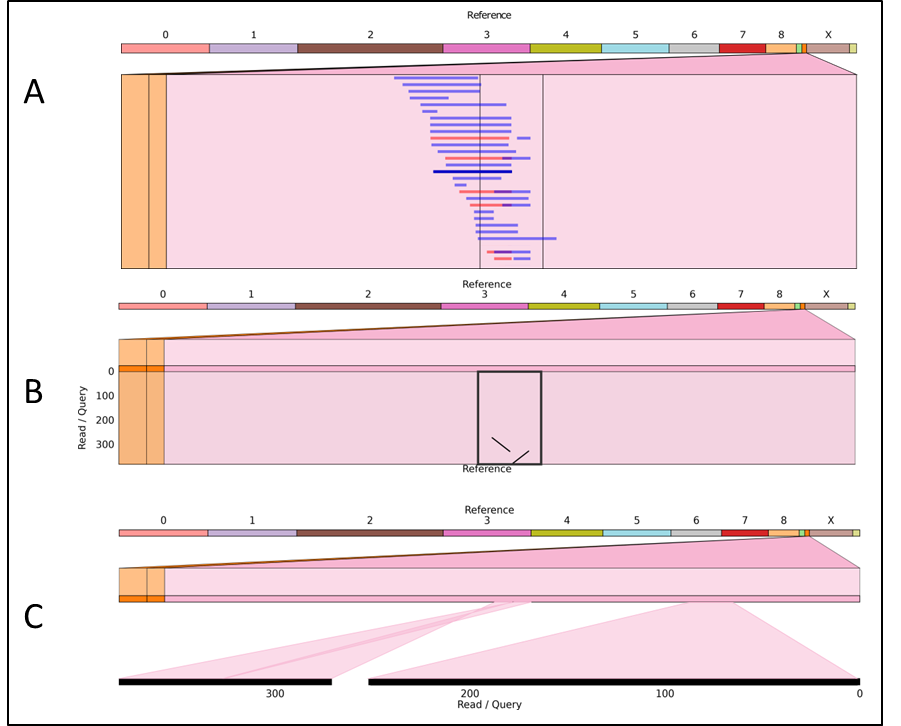


**Supplementary Figure 6|** Ribbon plot of Illumina bwa mem alignment from the pGMX vector|vector junction (12,695-><-12,756) in the AE54SL cell line (RGEN-D protocol). This is a single variant ‘head-to-head’ pGMX fusion in the AE54SL genome (*A*). This variant is captured in several read pairs, one of which is shown in *B* as a dot plot and in *C* as a ribbon plot.


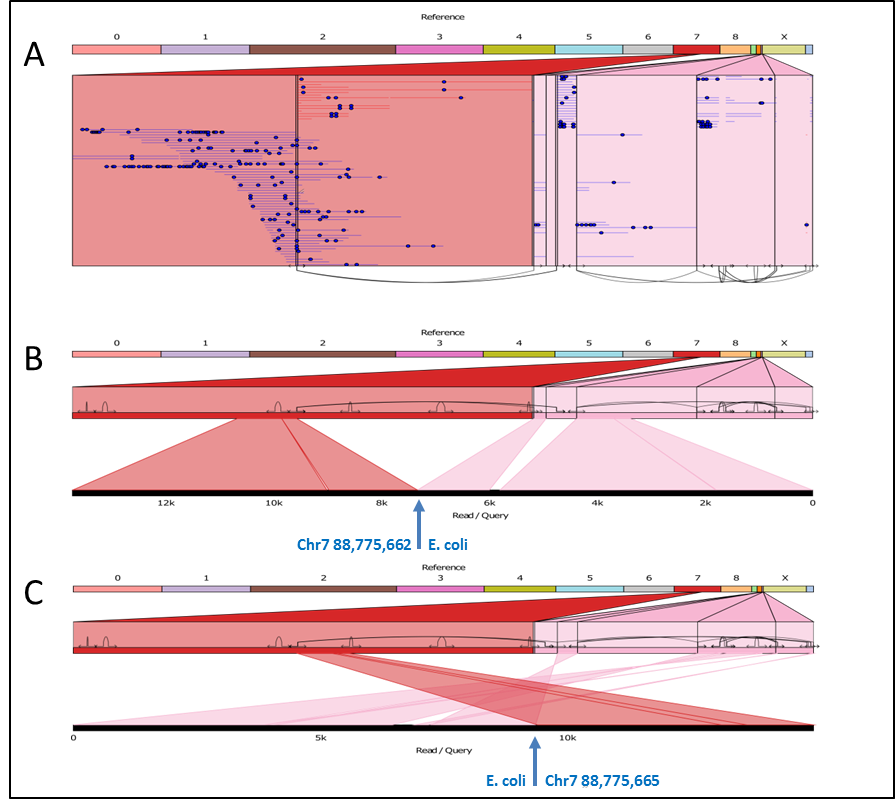


**Supplementary Figure 7|** Ribbon plots of PacBio NGM-LR alignments from a chromosome 7|E. coli-vector insert integration called by Sniffles (chr7 upstream breakpoint) in the AD49ZG. ***A***, the multi-read view of the regions involved in this fusion. The Sniffles calls are shown as connecting lines underneath in black; NGM-LR alignments are drawn in blue (forward) and red (reverse), where direction is with respect to the alignments at the breakpoints. ***B****,* ***C*** are two single-read examples supporting this integration event.


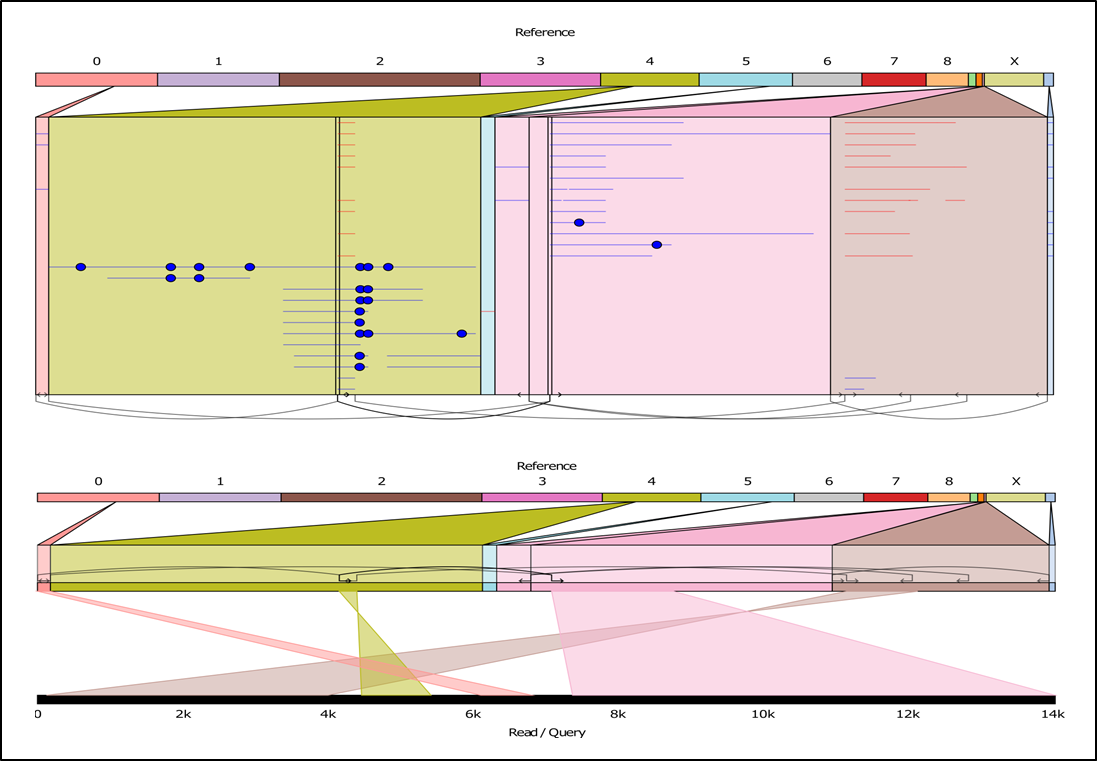


**Supplementary Figure 8|** Ribbon plots of PacBio NGM-LR alignments from a “3-hop” genomic fusion in the AD49ZG (top panel). This fusion has been created by merger of E. coli, CLE209 vector genomic fragments, and small fragments of chr1 and 4 within the ~349.3 Kbp integration site. These variants are captured together in several individual SMRT sequencing reads, one of which is shown in the lower panel.


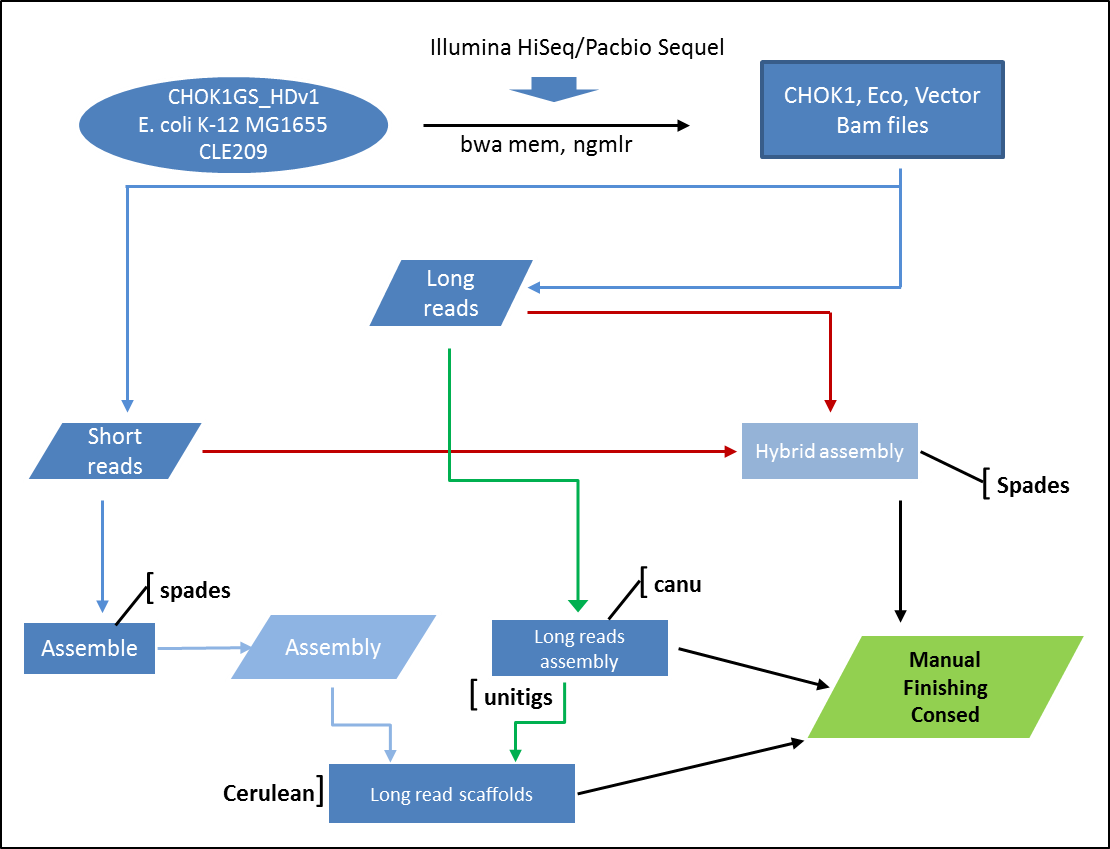


**Supplementary Figure 9|** A workflow for assembling an integration site in chromosome 7. A subset of short paired Illumina and long PacBio reads for the insert assembly was created by selecting reads that are mapped to the *E. coli* genome and CLE209 vector. Illumina and PacBio reads are assembled separately by SPAdes^8^ and Canu^9^, respectively. By default, Canu corrects the long PacBio reads, then trims the reads, and finally assembles them to unitigs. In addition, SPades directly utilizes short and long reads as inputs to generate a hybrid assembly. Cerulean^10^ is a scaffolder that uses long read information to scaffold pre-assembled contigs constructed from short reads. Finally, scaffolds and contigs from hybrid assembly are imported into Consed^11^ for manual curation of sequences and assembly them into a single contig via balancing the read coverage along the sequences.


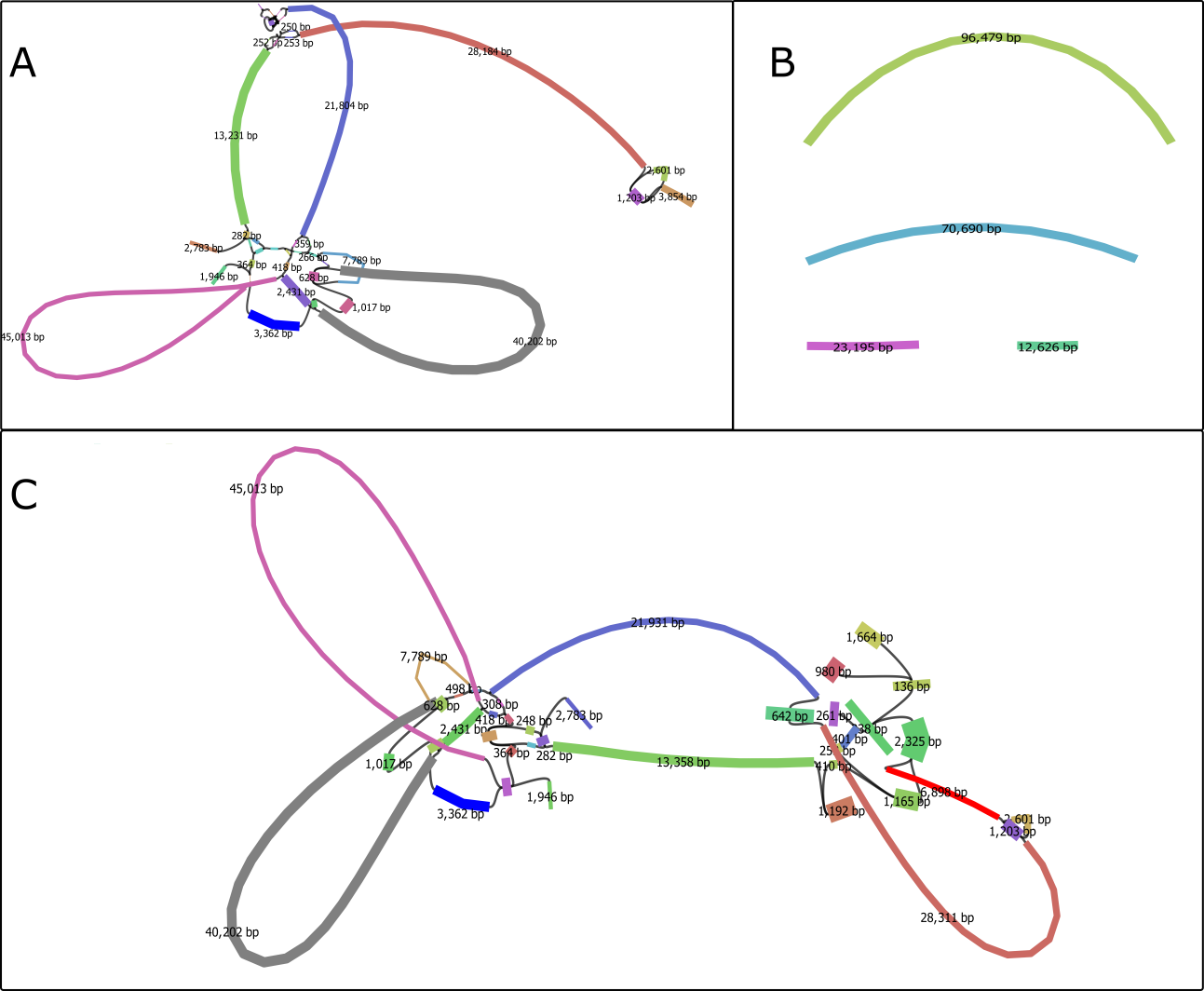


**Supplementary Figure 10|** Bandage^12^ plots of the AD49ZG chromosome 7 insert assembly produced by Spades and Canu. Spades and Canu GFA output localizes complex repeat regions, allowing for improved scaffolding. ***A***, Bandage plot of the insert assembly produced by Spades using only Illumina HiSeq2500 reads. ***B***, Bandage plot of the insert assembly produced by Canu using only PacBio reads. ***C***, Bandage plot of the hybrid assembly with Illumina and PacBio reads produced by SPades. Nodes are contigs sized by length and thickness (illumine read coverage), and edges indicate unused overlaps between contigs. A low coverage of the insert by PacBio reads results in a simple edge-free plot produced by Canu in ***B***. The large shared contigs in ***A***, ***C*** have the same colors, while small contigs are colored randomly and labeled with their length. The “thick” contigs in ***A***,***C*** were treated as duplicates when manually finishing the insert assembly in Consed.

**
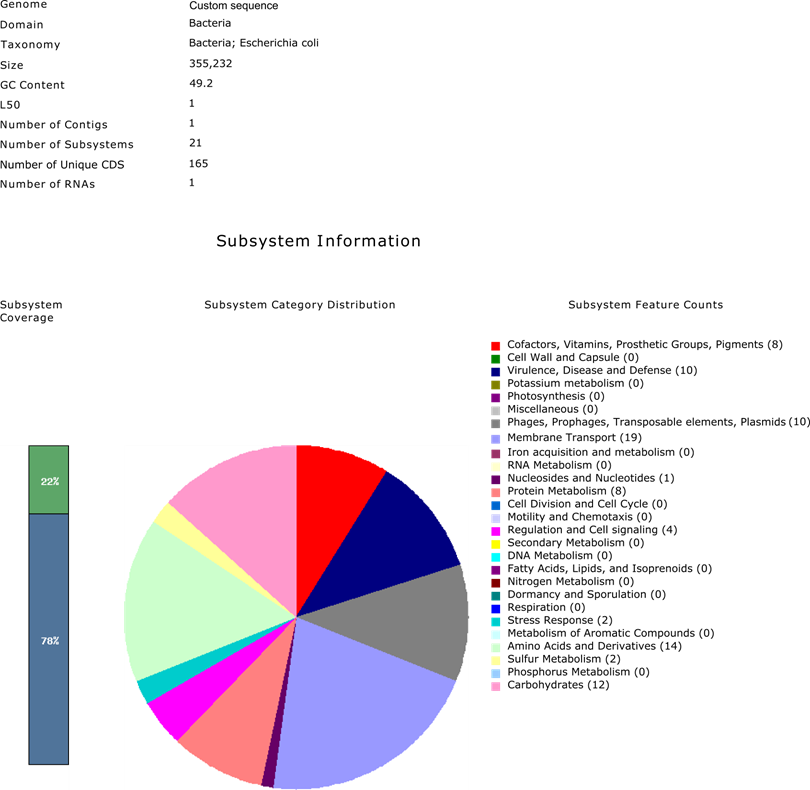
**

**Supplementary Figure 11|** Annotation summary of the E. coli|CLE209 vector insert in the AD49ZG genome. Annotation was done using Rapid Annotation using Subsystem Technology (RAST) server^13^ ([http://rast.nmpdr.org](http://rast.nmpdr.org/)). The annotation details are in the Supplementary Table 3.

**
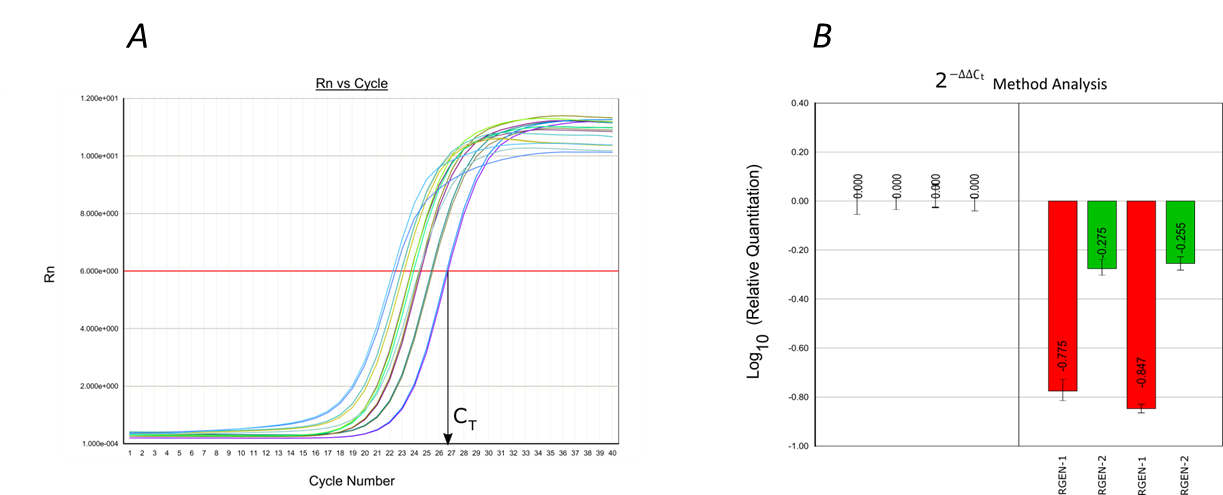
**

**Supplementary Figure 12|** Relative quantitation of gDNA digestion by RGENs. *A*, QPCR output, calculation of C_T_. Data is presented from the AS49ZH real-time PCR output. The PCR was run for 40 cycles. The point at which the curve intersects the threshold (horizontal red line) is the C_T_. *B*, RQ values. Fold-expression changes are calculated using the equation$2^{-\Delta\Delta Ct}$. Red/green bar pairs correspond to the sgRNA-1 and sgRNA-2 based RGENs with Cas9 protein from two different suppliers. QPCR outputs for untreated gDNA are used as calibrators, so that their RG values are set to 1.

**Supplementary References**

1. Brinkrolf, K. *et al.* Chinese hamster genome sequenced from sorted chromosomes. *Nat Biotech* **31**, 694–695 (2013).

2. Cricetulus griseus - Ensembl genome browser 92. Available at: https://useast.ensembl.org/Cricetulus_griseus_chok1gshd/Info/Annotation. (Accessed: 2nd May 2018)

3. Darling, A. C. E., Mau, B., Blattner, F. R. & Perna, N. T. Mauve: Multiple Alignment of Conserved Genomic Sequence With Rearrangements. *Genome Res* **14**, 1394–1403 (2004).

4. Guy, L., Roat Kultima, J. & Andersson, S. G. E. genoPlotR: comparative gene and genome visualization in R. *Bioinformatics* **26**, 2334–2335 (2010).

5. Talevich, E., Shain, A. H., Botton, T. & Bastian, B. C. CNVkit: Genome-Wide Copy Number Detection and Visualization from Targeted DNA Sequencing. *PLOS Computational Biology* **12**, e1004873 (2016).

6. Xie, C. & Tammi, M. T. CNV-seq, a new method to detect copy number variation using high-throughput sequencing. *BMC Bioinformatics* **10**, 80 (2009).

7. Nattestad, M., Chin, C.-S. & Schatz, M. C. Ribbon: Visualizing complex genome alignments and structural variation. *bioRxiv* 082123 (2016). doi:10.1101/082123

8. Bankevich, A. *et al.* SPAdes: A New Genome Assembly Algorithm and Its Applications to Single-Cell Sequencing. *J Comput Biol* **19**, 455–477 (2012).

9. Koren, S. *et al.* Canu: scalable and accurate long-read assembly via adaptive k-mer weighting and repeat separation. *Genome Res* **27**, 722–736 (2017).

10. Deshpande, V., Fung, E. D. K., Pham, S. & Bafna, V. Cerulean: A Hybrid Assembly Using High Throughput Short and Long Reads. in *Algorithms in Bioinformatics* 349–363 (Springer, Berlin, Heidelberg, 2013). doi:10.1007/978-3-642-40453-5_27

11. Gordon, D. & Green, P. Consed: a graphical editor for next-generation sequencing. *Bioinformatics* **29**, 2936–2937 (2013).

12. Wick, R. R., Schultz, M. B., Zobel, J. & Holt, K. E. Bandage: interactive visualization of de novo genome assemblies. *Bioinformatics* **31**, 3350–3352 (2015).

13. Aziz, R. K. *et al.* The RAST Server: Rapid Annotations using Subsystems Technology. *BMC Genomics* **9**, 75 (2008).
